# The Book World of Medicine and Science

**Published:** 1895-03-23

**Authors:** 


### THE BOOK WORLD OF MEDICINE AND SCIENCE.

THE STUDENTS' DICTIONARY OF MEDICINE, AND THE ALLIED SCIENCES. By ALEXANDER DUANE, M.D. (London: Longmans, Green, and Co. Philadelphia: Lea Brothers and Co. 1894. Price 21s.)

This is more than a mere etymological dictionary of medical terms. The derivation is given in cases where it is thought desirable, but, as the dictionary contains a large number of words common to the English language, and only special to medicine so far as a medical meaning has been attached to them, etymology is not entered into in every case. What, perhaps, will make the work most interesting to the student or the general reader is that, in addition to the mere definition, there is added to those words which denote diseases a short sketch of the symptoms and treatment, and a list of the varieties and complications which are commonly recognised. This is not a feature which will be of much advantage to the educated medical man, although, perhaps, it may make the book a little more amusing; but, after all, a dictionary is more for those who are lacking than for those who are full of knowledge, so these items may serve a purpose. There are some points to which we may refer as showing how this dictionary steps out of the ordinary path for the sake of supplying useful information. Under the head of poison there is a table giving the symptoms and the antidotes of those most commonly met with. Under artery there is a list of all arteries, except the smallest, giving their origin, and their lateral and terminal branches, with the distribution of each; nerve and muscle are dealt with in the same way; under exanthemata we find a table giving various details as to the eruptive fevers; bacteria gives us pages of details as to different micro-organisms and the diseases produced by them; and so on as regards many other topics. A study of the bewildering pages giving, under weight, a comparison, running to many decimals, of the various measures of weight and capacity in common use, ought to convince any one of the urgent necessity for a universal acceptance of the decimal system. A leading feature of this dictionary is the pronunciation, which is given for most words. This is always a difficulty, and we doubt whether any very good purpose is served by it. "Mawrg," for morgue, is interesting, as showing that another view is possible of almost anything, but does not appear very useful. "Kon-tay" jee-os' i-tee" is the pronunciation of a word synonymous with contagiousness; and here we would suggest that the double e, pronounced like ee in meet, does not at all accurately represent the sound of a final y, as it is made to do throughout the book. "Sil' ee-er-ee," for ciliary, may be right, but surely not if the ee is also right in "Koh-ree'ah," for chorea; in "ree-om'e-ur," for rheometer; or in "naf'tha-leen," for naptha-lene. Ascites, we observe, is to be pronounced "a-sey'teez," with no alternative, to which, we fancy, some will take objection. We do not say that this dictionary is perfect, and the very tables and other forms of current information, which will to some be an attractive feature, have in them an element of the ephemeral, nevertheless it will be very useful to many as a reference book. The worst of a dictionary is that it tends to fix permanently in the language words, or a use of words, which had better die out. The word quiz, for example, has as its essence the idea of jest or banter, and, commonly as we believe it is misused in America, it seems a pity to attach to it as a permanency the definition of "systematic instruction conducted by questions and answers, intended to prepare a student for examination," with the necessary riders of a "quiz-book" and a "quizzer or quiz-master," this being "one who conducts a quiz." The term is short, and may be expressive so far as banter and sarcasm are educational agencies, but it hardly sounds appropriate for a course of "systematic instruction."

